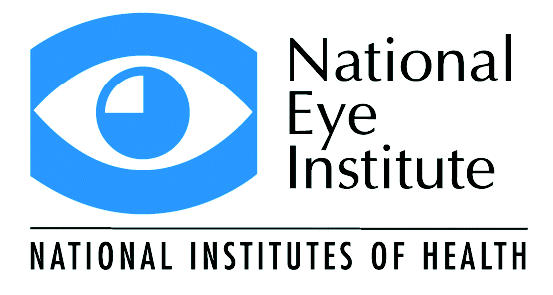# EHPnet: National Eye Institute

**Published:** 2005-12

**Authors:** Erin E. Dooley

The National Eye Institute (NEI) is the primary institute of the NIH for supporting and conducting research on preventing, diagnosing, and treating eye diseases and other vision disorders. Currently the NEI oversees approximately 1,600 research projects at more than 250 institutions in addition to the research ongoing at its own facilities in Bethesda, Maryland. The institute also works to translate research findings into clinical applications and to raise public awareness about eye and vision problems. The NEI uses its website, located at **http://www.nei.nih.gov/**, to help disseminate information about its many programs.

The What’s New section provides links to newly released NEI-funded research and other topics of interest to those in the field. The more in-depth News and Events section includes press releases, clinical alerts for professionals, information on meetings and special events, and a list of official statements and reports on vision.

The Health Information page links to information on 21 eye diseases and disorders. There is also a section on basic eye anatomy with diagrams of the eye, links to glossaries of eye terminology, a collection of eye care resources, NEI information provided in Spanish, and a way to order NEI materials online. The collection of eye care resources consists of an eye health organizations database; a page of frequently asked questions about clinical trials and how they are conducted; and tips on finding an eye care professional, procuring financial assistance for eye care, and talking to doctors about eye health.

More information on NEI clinical trials is available on the Research Funding page and through the Clinical Studies Database. The Research Funding page has information on grant and funding opportunities for researchers, news on staff appointments, updates on grants and funding policies, and overviews of councils and workshops of interest to NEI researchers, among other resources. The Clinical Studies Database provides a list of all ongoing and completed NEI-supported studies. This section also includes study results and lists of journal articles that have been generated by the research, as well as a list of NEI studies that are currently enrolling participants. Site visitors can search the database for studies under six topic areas or by keyword, study location, age of study participants, patient recruitment status, or study status.

The Education Programs page offers overviews of NEI outreach activities. Through the National Eye Health Education Program, the NEI conducts large-scale public and professional educational activities in partnership with national organizations. Specialty initiatives within this program focus on diabetic eye disease, glaucoma, low vision (when everyday tasks become difficult to do even with corrective lenses, medicine, or surgery), and educating Spanish-speaking Americans about eye and vision problems. VISION is a teaching supplement for grades 4 through 8 that is available for download at no charge. This 16-page guide helps teachers plan lessons about how the eye works, eye problems, and eye safety. The supplement was developed in cooperation with the Association for Research in Vision and Ophthalmology. THE EYE SITE is an NEI-sponsored exhibit that travels to shopping malls around the United States to educate the public about low vision, vision rehabilitation services, and vision adaptive devices, as well as about the NEI itself. The exhibit features five colorful kiosks and an interactive multimedia program. Another exhibit, VISION, educates visitors to science museums about how vision works and about how researchers are working to develop ways to protect our eyes from disease and developmental problems.

## Figures and Tables

**Figure:**